# Pharmacokinetics/pharmacodynamics of chloroquine and artemisinin-based combination therapy with primaquine

**DOI:** 10.1186/s12936-019-2950-4

**Published:** 2019-09-23

**Authors:** André Daher, Ghait Aljayyoussi, Dhelio Pereira, Marcus V. G. Lacerda, Márcia A. A. Alexandre, Cristiana T. Nascimento, Júlio Castro Alves, Laís Bastos da Fonseca, Diego Medeiros Dias da Silva, Douglas Pereira Pinto, Danielle Fonseca Rodrigues, Ana Carolina Rios Silvino, Taís Nóbrega de Sousa, Cristiana Ferreira Alves de Brito, Feiko O. ter Kuile, David G. Lalloo

**Affiliations:** 1 Institute of Drug Technology (Farmanguinhos), Oswaldo Cruz Foundation (FIOCRUZ), Rio de Janeiro, Brazil; 2 Vice-presidency of Research and Biological Collections, Oswaldo Cruz Foundation (FIOCRUZ), Rio de Janeiro, Brazil; 3 Liverpool School of Tropical Medicine, Liverpool, UK; 4 Tropical Medicine Research Center of Rondonia (CEPEM), Porto Velho, Rondonia Brazil; 5 Federal University of Rondonia (UNIR), Porto Velho, Rondonia Brazil; 6 Research Institute Leônidas & Maria Deane, FIOCRUZ, Manaus, Brazil; 7 Foundation of Tropical Medicine Dr. Heitor Vieira Dourado, Manaus, Brazil; 8 National Institute of Infectious Disease, Oswaldo Cruz Foundation (FIOCRUZ), Rio de Janeiro, Brazil; 9 Laboratory of Pharmacokinetics (SEFAR), Oswaldo Cruz Foundation (FIOCRUZ), Rio de Janeiro, Brazil; 10 Institute René Rachou, Oswaldo Cruz Foundation (FIOCRUZ), Belo Horizonte, Brazil

**Keywords:** Malaria, *Plasmodium vivax*, Anti-malarial treatment, Chloroquine, Mefloquine, Lumefantrine, Primaquine, Artemisinin-based combination therapy, ACT, Pharmacokinetics, Clinical trial

## Abstract

**Background:**

Activation of hypnozoites of vivax malaria causes multiple clinical relapses, which contribute to the *Plasmodium vivax* burden and continuing transmission. Artemisinin-based combination therapy (ACT) is effective against blood-stage *P. vivax* but requires co-administration with primaquine to achieve radical cure. The therapeutic efficacy of primaquine depends on the generation of a therapeutically active metabolite via cytochrome P450 2D6 (CYP2D6). Impaired CYP2D6 metabolism has been associated with primaquine treatment failure. This study investigated the association between impaired *CYP2D6* genotypes, drug-exposure to the long-acting ACT component (schizonticidal drugs) and tolerance and efficacy.

**Methods:**

Adult patients with acute vivax malaria were enrolled in a recently completed trial and treated with artesunate–mefloquine, chloroquine or artemether–lumefantrine. All received concomitant primaquine (0.5 mg/kg/day for 7–9 days). The association between efficacy and safety and drug exposure was explored using area-under-the-curve (AUC) and half-life (t_1/2_) estimates obtained by non-compartmental analysis of the long half-life drugs. Parasite recurrences by day 63 were categorized as related relapses or re-infections/unrelated hypnozoite activation by genotyping three microsatellite loci and two polymorphic loci of merozoite surface antigen-1. The *CYP2D6* genotype was identified with Taqman assays by real-time PCR to 9 polymorphisms (8 SNPs and one deletion). Impaired CYP2D6 activity was inferred using the Activity Score System.

**Results:**

Most recurrences in the ASMQ (67%), CQ (80%) and AL (85%) groups were considered related relapses. Eight of nine (88.9%) of the patients with impaired CYP2D6 activity relapsed with related parasite compared to 18/25 (72%) with normal activity (RR = 1.23, 0.88; 1.72, p = 0.40). There were no associations between the measured PK parameters and recurrence. Patients with longer chloroquine half-lives had more pruritus (RR = 1.09, 1.03; 1.14, p = 0.001). Higher CQ AUCs were associated with reduced falls in haemoglobin by day 14 (Coef − 0.02, − 0.005; − 0.03, p = 0.01). All regimens were well tolerated.

**Conclusion:**

Genotyping of *P. vivax* showed that activation of related (homologous) hypnozoites was the most frequent cause of recurrence. The high proportion of the impaired CYP2D6 activity among patients with recurrent infections suggests that slow primaquine metabolism might influence related relapse rates in Brazil among patients receiving primaquine for radical cure, although confirmatory studies are needed. There was no association between drug exposure of the long-acting ACT component (schizonticidal drugs) and risk of related relapse. ACT was well tolerated. These results provide further re-assurance about the safety and efficacy of ACT when combined with short course primaquine to treat uncomplicated malaria vivax in Brazil.

*Trial registration* RBR-79s56s (http://www.ensaiosclinicos.gov.br/rg/RBR-79s56s/)

## Background

The biological features of vivax malaria provide major challenges for pre-elimination and elimination programmes in areas co-endemic for *Plasmodium falciparum* and *Plasmodium vivax* [1]. These features include the early appearance of gametocytes, a high proportion of asymptomatic or chronic carriers [2] and the parasite latent form hypnozoites that produce relapses. Relapses make a major contribution to the global *P. vivax* burden [3]. It was estimated that relapses constituted 76–90% and 79% of total infections in Papua New Guinea and Thailand, respectively [4]. To eliminate this reservoir of latent infections, simple effective radical treatment of vivax using 8-aminoquinolines, such as primaquine or the recently approved analogue tafenoquine, is required.

In most countries with endemic *P. vivax,* the preferred first-line radical treatment for *P. vivax* remains chloroquine, combined with 7- to 14-day primaquine regimens, and this has barely changed since the 1950s [5]. Chloroquine is no longer recommended for the case management of falciparum malaria due to the spread of parasite resistance [6], resulting in the use of different treatment regimens for *P. vivax* and *P. falciparum* in areas where these species are co-endemic. This is programmatically complicated. The use of a single regimen to treat all species of malaria would simplify malaria treatment guidelines [7]. Artemisinin-based combination therapy (ACT) is emerging as the best option in this context, particularly in settings where there are concerns about chloroquine-resistant *P. vivax* [8]. ACT is effective against the blood stage of *P. vivax* [9], but must be co-administered with primaquine to eliminate *P. vivax* hypnozoites [8, 10]. Short-course primaquine regimens are preferable as they have been proven to have an efficacy not inferior to the standard 14 days’ regimens [11–14]. However, there is a relative lack of data on the safety, pharmacodynamics and pharmacokinetics of ACT when provided in combination with daily primaquine regimens for the radical cure of *P. vivax* [15]. Safety is a major concern when deploying new treatments, but there are also concerns that primaquine/ACT drug interactions may reduce the overall regimen efficacy by inhibiting CYP2D6 or reducing synergistic effect of the current regimen. There are uncertainties about the best partner ACT, as drugs with a longer half-life may prevent early relapses.

Characterization of parasites and patients’ drug metabolism is needed to ascertain the pharmacodynamics, pharmacokinetics and therapeutic success of these regimens. The main malaria clinical trials outcomes are parasitological clearance and recurrence rate [16]. Vivax recurrence includes: (i) recrudescence of parasites that have been previously cleared and microscopically undetectable; (ii) re-infection from another mosquito bite; and, (iii) relapses, i.e., activation of hypnozoites, genetically related (homologous) or unrelated (heterologous). The primaquine metabolite that is active against human hypnozoite is unknown [17], but the metabolism of primaquine to its active metabolite is dependent on the cytochrome P450 enzyme CYP2D6 [17–19]. Low *CYP2D6* activity results in slow metabolism of primaquine to the active metabolite. CYP2D6 activity may be a proxy of primaquine’s active metabolite exposure and a risk factor for relapse among primaquine recipients [20–22]. Similarly, the cytochrome P450 enzymes CYP2C8 was investigated as it is known to participate in the metabolism of chloroquine [23].

A previous randomized clinical trial in Brazil of the treatment of uncomplicated vivax malaria compared the safety and efficacy of the fixed-dose ACT artemether–lumefantrine and artesunate–mefloquine against the standard treatment with chloroquine, all three in combination with short-course primaquine (0.5 mg/kg/day for 7–9 days) [24]. This current study investigated the pharmacokinetics of the long-acting ACT component (schizonticidal drugs) with concomitant primaquine upon the safety and efficacy of these three treatment regimens and the influence of genetic variability of parasite and host, including the frequency of mutations in CYP2D6 gene over relapse rate.

## Methods

### Overview study design

The patients included in the current analysis were enrolled in a larger clinical trial previously published and designed in accordance with WHO guidelines [16]. The trial was designed to evaluate the safety and efficacy of the schizonticidal drugs with concomitant use of primaquine for vivax cure. The details of the trial design and methods have been reported elsewhere [24]; in brief, patients were eligible if they had acute uncomplicated malaria due to *P. vivax* mono-infection confirmed by microscopy, with fever or a history of fever in the previous 48 h, were aged 18 to 70 years old, weighed between 50 and 90 kg, and had parasite densities > 250/μL and haemoglobin levels > 7.0 g/dL. G6DP deficiency was not an exclusion criterion. They were randomly allocated to three treatment groups: (a) artesunate–mefloquine (100 + 200 mg QD for 3 days) (ASMQ); (b) chloroquine (CQ) (600 mg on day 1, and 450 mg on days 2 and 3); and, (c) artemether–lumefantrine (20 + 120 mg BID for 3 days) (AL). All three arms received the same concomitant primaquine regimen (7–9 days: 0.5 mg/kg/day).

Patients were assessed on the day of enrolment and days 1, 2, 3, 7, 14, 21, 28, 42, and 63. The main endpoints were either treatment failure or adequate clinical and parasitological response. Blood samples were collected for parasite counts at every scheduled visit, on any day of treatment failure and for drug levels on days 0, 3, 7, 14, 21, 28, 42, and 63. Samples (100 μL) were transferred to Whatman (USA) ET 31 CHR E 3MM filter papers for later pharmacokinetic analysis and parasite genotyping [25].

Parasitological densities were estimated using Giemsa-stained blood slides at a magnification of 1000× using WHO-recommended methods [16]. Adverse events (AE) were assessed at each follow-up visit, and patients were encouraged to return to the clinic if they were ill in between scheduled visits. All AEs, including laboratory abnormalities, were categorized by body system.

### Pharmacokinetics/pharmacodynamics

Whole blood concentrations of mefloquine (MQ), chloroquine (CQ) and lumefantrine (LMF) were measured using a validated HPLC-MS/MS method in accordance with Brazilian [26] and international regulatory requirements for bio-analytical methods [27]. The pharmacokinetics assays were conducted at the Equivalence and Pharmacokinetics Service (SEFAR)/Oswaldo Cruz Foundation, which is accredited by the Brazilian regulatory agency, Agência Nacional de Vigilância Sanitária (ANVISA). Non-compartmental analysis was performed for CQ, LMF and MQ using the Pmetrics^®^ [28] package to estimate two main parameters; the overall area under the curve (AUC)_(3–63 days)_ and the terminal elimination half-life (t_1/2_). The terminal elimination half-life was only calculated for subjects with five or more available samples. These two parameters were used as proxy indicators for overall drug exposure for correlation analyses with drug efficacy and safety profiles.

The correlation between drug exposure and treatment failure was evaluated using the Mann–Whitney test. The effect of pharmacokinetic parameters on the frequency of AEs likely or probably related to the test drug was expressed as the relative risks (RR) obtained from random effects generalized estimation equation (GEE) log-binomial regression models. Linear regression was used to test the effects of PK parameters on the fall in haemoglobin (Hb) concentrations by day 14 relative to enrolment values [29]. The effects of these pharmacokinetic parameters on treatment failure (both early and later failures) were evaluated as odds ratios (OR) from binomial logit link regression models, using generalized linear models (GLM). Time to failure was tested using standard Cox regression. The proportion of clonal variability at recurrence was compared between treatment arms using Fisher’s exact test. Two-sided p-values of < 0.05 were considered statistically significant.

### DNA extraction and genotyping of parasites microsatellites and polymorphic blocks of MSP-1 and patients’ CYP2D6 and CYP2C8

DNA was extracted from dried blood using QIAamp DNA blood mini kit (Qiagen, Hilden, Germany) following the instructions of the manufacturer. Three microsatellite loci (MS2, MS6, MS7) and two polymorphic loci (blocks 2 and 10) of Merozoite surface antigen 1 (MSP-1) were amplified using specific primers and conditions as previously described [30, 31]. The exact length and relative abundance (fluorescence levels) of each PCR product were determined in the DNA automatic sequencer (ABI 3730, Applied Biosystems, Thermo Fischer Scientific, Waltham, MA, USA) with fluorescein-labelled forward primers and an internal size standard (GeneScan 500 LIZ, Applied Biosystems). The predominant allele for each locus was identified as the highest peak of fluorescence in the electropherogram using GeneMapper 4.1 software (Applied Biosystems). The multiplicity of parasite variants was estimated measuring extra peaks in the electropherogram with fluorescence above the cut-off (150 arbitrary fluorescence units) and at least one-third the high of the main peak. Parasite recurrences within 63 days were categorized as ‘related’, including totally identical (homologues) if all five polymorphic loci (MS2; MS6; MS7; MSP1B2; MSP1B10) were identical and ‘similar’ if 80% of their alleles were identical; and otherwise as unrelated (heterologous) (Additional file 1). Number of alleles and heterozigosity expected were calculated in Arlequim software v. 3.5.2.2 (http://cmpg.unibe.ch/software/arlequin35/).

The cytochrome P450 enzymes CYP2D6 [17, 19] and CYP2C8 [23] are known to participate in the metabolism of the primaquine and chloroquine, respectively. Two SNPs (G416A[rs11572080] and A805T [rs11572103]) were genotyped in the *CYP2C8* gene. In the *CYP2D6* gene eight SNPs were genotyped; (G-1584C [rs1080985], C100T [rs1065852], C1023T [rs28371706], G1846A [rs3892097], C2850T [rs16947], G2988A [rs28371725], G3183A [rs59421388] and G4180C [rs1135840]) and one deletion (2615-2617delAAG [rs5030656]) were genotyped. The copy number was also determined. All SNPs genotyping were performed by real-time PCR using specific hydrolysis probe [22] in ViiA 7 Real-time PCR system (Applied Biosystems). Haplotypes and *CYP2D6* star alleles were inferred using the Phase software (version 2.1). *CYP2D6* gene copy number was determined with Hs00010001_cn assay (Applied Biosystem) in Real-time PCR [20]. Each allele got one value that was used to calculate the CYP2D6 activity score (AS). Patient were categorized based on their AS score into normal metabolizer fast (gNM-F) (AS = 1.5 or 2.0), normal metabolizer slow (gNM-S) (AS = 1), intermediate metabolizer (gIM) (AS = 0.5), poor metabolizer (gPM) (AS = 0), and ultra metabolizer (gUM) (more than 2 copies of the normal allele) (AS ≥ 2.0). Impaired CYP2D6 activity was defined as AS scores less than 1.5 (Additional file 2).

## Results

### Population

The study is based on a sub-set of samples from the original trial. Only 2/3 of samples (1400 samples of 175 patients) were evaluated in pharmacokinetics/pharmacodynamics analysis due to logistical issues, however the baseline characteristics of this sub-set were similar across the three arms (Table 1). Characterization of parasites and drug metabolism was conducted in all 35 patients with parasite recurrence within 63 days.

**Table 1:** Baseline characteristics of the study population

	ASMQ	CQ	AL
N of patients	60	58	57
Male n (%)	35 (58)	46 (79)	39 (68)
Weight in kg	70 (11)	73 (10)	73 (10)
Haemoglobin in g/dL	13.4 (1.8)	14.0 (3)	13.6 (2)
Parasitaemia/μL^a^	2145.56 [258–13,335]	2155.78 [285–17,685]	2444.71 [270–19,815]
Age in years	39.0 (11)	42.1 (10.5)	38.7 (11)

Data represent n (%) and means and (SD)

^a^Parasitaemia was expressed by geometric mean and [min–max]

### Characterization of parasites and drug metabolism in the population with treatment failure

The frequency of related (homologous and similar) and unrelated (heterologous) parasites among 35 patients with parasite recurrence within 63 days by study arm is shown in Table 2. Overall, 67, 80 and 85% of the recurrent malaria in the ASMQ, CQ and AL groups, respectively, were considered related relapses. Among the recurrences the pooled percentage of related relapses across the three arms was 77.1%.

**Table 2:** Clinical outcomes aggregated by drug treatment (%)

Clinical outcome (%)	PCR results	ASMQ		CQ		AL		Overall	
Related relapse	Homologous	6	66.7%	6	80.0%	8	84.6%	20	77.1%
	Similar	2		2		3		7	
Reinfections or unrelated hypnozoites’ activation	Heterologous	4	33.3%	2	20.0%	2	15.4%	8	22.9%
Overall		12		10		13		35	

Genetic analysis of the parasite populations comparing all recurrences also demonstrated that 77.1% (27/35) of patients presented with a single clonal infection at the initial infection and at the recurrence. The number of parasite variants between initial infection and recurrence remained the same in 23 (65.7%) of the 35 patients with recurrent infections and increased in 10 (28.5%) and decreased in 2 (5.7%). There was no difference in multiplicity of parasite clones at recurrence between treatment arms (p = 0.51).

### Cyp2d6

Eight of 9 (88.9%) patients classified to have reduced enzymatic activity for primaquine metabolism based on their CYP2D6 genotypes relapsed with related parasites (RR = 1.23 95% CI (0.88–1.72) p = 0.40) (Table 3). Eighteen out of 25 (72%) normal metabolisers had related relapses.

**Table 3:** Inferred phenotype of CYP2D6 based on genotyping by treatment arm (%)

CYP2D6 metabolism	AS	Phenotype	ASMQ		CQ		AL^a^	
Normal	1.5 or 2	gNM-F	5	50.0%	9	90.0%	9	81.8%
	≥ 2	gUM	1		0		0	
Impaired	1	gNM-S	5	50.0%	0	10.0%	2	18.2%
	0.5	gIM	0		1		0	
	0	gPM	1		0		0	

*AS* activity score, *gNM-F* normal-fast metabolizer, *gUM* ultrarapid metabolizer, *gNM-S* normal-slow metabolizer, *gIM* intermediate metabolizer, *gPM* poor metabolizer

^a^CYP2D6 phenotype of two patients (ID 33 and 231) could not be defined

### Cyp2c8

The frequency of two polymorphisms in *CYP2C8* gene in the population who failed is presented in Table 4. Out of the 10 patients in the CQ arm with parasite recurrence, one had the SNP G416A genotype indicative of reduced enzyme activity of *CYP2C8*. The AUC and half-life for chloroquine of this patient were 91.9 µg/mL h and 11.32 days, respectively, compared with 102.3 µg/mL h and 19.3 days in the 9 CYP2C8 non-mutated genotypes in the CQ arm. These results for the patient with the mutated genotype fall within the 95% CI for the overall population (Table 5).

**Table 4:** Inferred phenotype of CYP2C8 based on genotyping by drug treatment group

CYP2C8 (SNP)	Genotype	MQ	CQ	LMF
G416A	Normal	7	9	13
	Mutant^a^	5	1	0
A805T	Normal	12	10	13
	Mutant	0	0	0

^a^All mutants are heterozygotes

**Table 5:** Median of pharmacokinetic parameters for mefloquine, chloroquine and lumefantrine in patients with homologous parasites by treatment outcome

	Mefloquine			Chloroquine			Lumefantrine		
	Cure	Relapse	p-value	Cure	Relapse	p-value	Cure	Relapse	p-value
N of patients	53	6	–	52	7	–	52	7	–
AUC_(0–63 day)_ (µg/mL h)									
Per outcome	338.6	302.0	0.67	105.9	95.8	1.0	7.3	3.73	0.96
Overall	338.6 [256.0–408.0]		–	103.8 [83.0–126.3]		–	6.7 [2.7–12.0]		–
Half-life (days)									
Per outcome	17.7	25.6	0.58	18.5	19.3	0.42	NA	NA	–
Overall	17.8 [15.0–24.8]		–	18.7 [15.4–27.8]		–	NA		–

Overall values are median with 25–75 percentiles in square brackets and N = 59

*NA* non-applicable

### Pharmacokinetics/pharmacodynamics

**Fig. 1:**
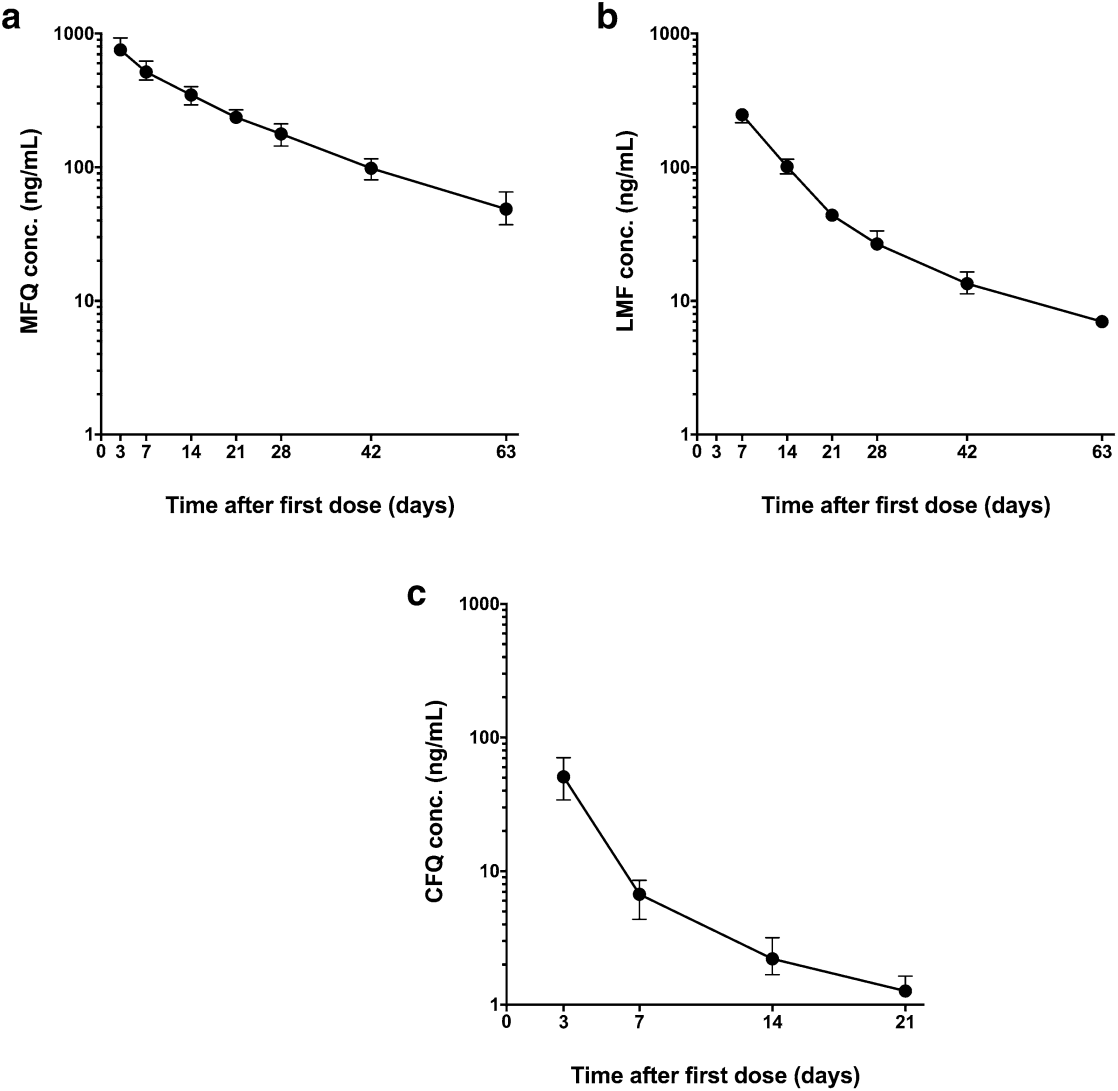
Pharmacokinetic profile of Mefloquine (MQ), Lumefantrine (LMF) and Chloroquine (CQ). Profiles were generated from 58 to 60 subjects for each drug. Data shows median with 95 confidence interval (CI). **a** Mefloquine concentration over the time, **b** lumefantrine concentration over the time and **c** chloroquine concentration over the time

AUC_0–63 day_ values were calculated for each patient in each arm; the terminal elimination half-life (t_1/2_) could only be calculated for those receiving CQ or MQ. Figure 1 shows the terminal PK profile generated for MQ, CQ and LMF.

Drug exposure (defined by AUC_3–63 day_ or elimination half-life) under the therapeutic threshold could potentially explain the relapses and recrudescence, but not re-infections. The relationship of drug exposure (defined by AUC_3–63 day_ or elimination half-life) to recurrence was investigated by comparing the PK parameters in the cured population with those with confirmed related relapse (homologous + similar). There were no statistically significant differences in AUC_3–63 day_ or elimination half-life for MQ, CQ or LMF between patients without recurrent infections by day 63 and those with relapses in univariate (Table 5). Weight and gender were also not associated with parasite recurrence by day 63 (Additional file 3), parasite clearance by day 3 (Additional file 4), or time to failure (Additional file 5).

Higher drug exposures could be associated with a higher frequency of AEs. The influence of AUC, half-life and weight on the frequency of AEs likely and probably related to the treatment per body systems (n ≥ 30) are presented in Additional file 6. The relative risk of having pruritus increased as the half-life of CQ increased (RR 1.09, 95% CI 1.03–1.14, p = 0.001). Conversely, the influence of drug exposure on the Hb drop (defined as Hb at day 14 − Hb at baseline/Hb at baseline) shown that the higher the AUC of CQ the lower the reduction in the Hb decline (Coef − 0.02, 95% CI − 0.03; − 0.00, p = 0.01) (Additional file 7). Please number in sequence i.e. first 6 then 7).

## Discussion

This study showed by genotyping polymorphic loci of *P. vivax* that relapse due to related parasites was the most frequent cause of recurrence by day 63 in all three treatment arms of the previous trial [32], where 13–16% of infections recurred by day 63. The current study also showed that the treatment failure rate in patients with reduced inferred CYP2D6 activity (26%) was higher than in the general population (≅ 11%) [33]. Moreover, 8 out of 9 recurrences among patients with low CYP2D6 activity were related relapses compared to 18 out of 25 with inferred normal CYP2D6 activity. Low CYP2D6 activity and presumptive low exposure to the active metabolite of primaquine or disruption of the PQ-enzyme interactions might influence related relapse rates in Brazil, although further studies are need to elucidate this effect. Likewise, the impaired activity of CYP2C8 may result in a lower exposure to desethylchloroquine, the chloroquine active metabolite. The PK parameters and clinical outcomes of the single patient with impaired CYP2C8 in CQ arm did not differ from the overall population.

Other causes of low exposure to primaquine are also potential risk factors for relapses, such as low adherence or impaired bioavailability. Similarly, others factors which affect the success of anti-infective therapeutics may influence the responses to the radical treatment, such as differences in the biology of the parasite, the immune status of the patients and the density of latent hypnozoites [34].

This was the first time that some of the ACT combinations, namely ASMQ, were evaluated for the cure of *P. vivax* in clinical trial conditions, and thus in combination with daily primaquine (0.5 mg/kg/day for 7–9 days) regimens. The correlation between drug exposure to the long-acting components of the ACT and the risk of recurrence and AEs was also assessed. Potential interactions between primaquine and the ACT, and also the safety of these regimens, have not been extensively assessed [15]. Drug interactions such as lumefantrine inhibiting CYP2D6 [35] may reduce the overall regimen efficacy; on the other hand, drugs with a longer half-life may prevent early relapses. This study could not demonstrate a significant associations between the PK parameters of the long half-life drugs and the risk of recurrence, or the risk of relapse due to either homologous and heterologous relapses or time to recurrence (Additional files 3, 4, 5), although the numbers of relapses were small. Patients with longer chloroquine elimination half-life estimates were more likely to report pruritus. Transient, mild to moderate pruritus is a well-known adverse effect of chloroquine [36] and a threat to treatment adherence. A smaller drop in haemoglobin by day 14 was associated with higher CQ exposure (AUC), which may reflect better therapeutic efficacy achieved with higher concentrations of CQ [37].

This study has several limitations. The blood sampling schedule was designed to evaluate the blood levels of the long half-life drugs; the pharmacokinetic data allowed the prediction of the drug exposures up to 63 days post-treatment with up to 8 sample points available for each patient. It did not allow a proper modelling of primaquine levels and limited the calculation of the elimination half-life of lumefantrine. The absence of desethylchloroquine blood levels measurement is another study limitation. A trial designed with 6 months follow-up would have been able to evaluate relapses with more accuracy, as the median time to vivax recurrence in Brazil is 71 days [13]. The characterization of parasites and drug metabolism were conducted only in 35 patients with treatment failure limiting the comparisons. Genotyping vivax parasites to infer relapse frequencies also presents limitations. Genotyping in vivax does not allow differentiation between new infections (reinfections) or activation of unrelated (heterologous) hypnozoites. In this study, only recurrences with homologous and similar parasites were considered relapses. However, relapses are often heterologous activation of hypnozoites [31, 38]. The recrudescence of sub-microscopic parasite population [39] is also a biologically plausible explanation for homologous parasites in two samples. Future use of more sensitive parasite detection strategies, such as ultrasensitive PCR of all consecutive samples of the patients who failed could elucidate these results.

## Conclusion

The genotyping of polymorphic loci of *P. vivax* showed that relapse due to genetically related parasites was the most frequent cause of recurrence in all three treatment arms. The high proportion of *CYP2D6* genetic polymorphisms among patients with recurrent infections suggests that impaired primaquine metabolism might influence the related relapse rates in Brazil among patients receiving primaquine for radical cure, further studies are needed to confirm this finding. The three ACT regimens were very effective, and there was no association between drug exposure levels of the long-acting components of the ACT and risk of relapse. The ACT was well tolerated overall. These results provided further reassurance about the safety of the combined use of ACT and short-course primaquine (0.5 mg/kg/day for 7–9 days) to treat uncomplicated malaria vivax in Brazil.

## Supplementary information

**Additional file 1.** Genotyping of *Plasmodium vivax* polymorphic loci from patients during initial infection and recurrence. Size of PCR products in base pairs (bp). ^#^Number of patient and day of sample collection: d0—diagnosis and treatment, dX—the day of recurrence. *Allele similar to initial infection present in lower intensity. *NA* non-amplified.

**Additional file 2.** Genotyping of CYP2C8 gene and CYP2D6 gene and predicted phenotype of CYP2D6 activity. a CYP2D6 haplotype inferred using Phase software. b Activity score and inferred phenotype of CYP2D6: AS = 1.5 or 2 − normal metabolizer fast (gNM-F); AS ≥ 2 − ultra metabolizer (gUM) (more than 2 copies of the normal allele); AS = 1 − normal metabolizer slow (gNM-S); AS = 0.5 − intermediate metabolizer (gIM); AS = 0 − poor metabolizer (gPM), according to Gaedigk et al. [20]. c Sum of AS attributed to allele 1 and 2 of CYP2D6 gene. *NP* not performed.

**Additional file 3.** Evaluation of pharmacokinetics’ parameters, gender and weight as predictors of failures per treatment drug (Generalized Linear Model, binomial logit link).

**Additional file 4.** Evaluation of pharmacokinetics’ parameters and weight as predictors of D3 failures per treatment drug (Generalized Linear Model, binomial logit link). Only available to chloroquine, all males. ASMQ and AL 100% presented clearance at D3.

**Additional file 5.** Evaluation of pharmacokinetics’ parameters, gender and weight as predictors of time to failures per treatment drug (Cox regression).

**Additional file 6.** Evaluation of pharmacokinetics’ parameters as predictors of frequent (n ≥ 30) adverse event (possible and likely related to treatment) per system and drug using Generalized Estimation Equation log-binomial regression. *CQ AUC and weigh correlation is significant at the 0.01 level (2-tailed). Weight was excluded as a covariate.

**Additional file 7.** Evaluation of pharmacokinetics’ parameters and weight as predictors of the drop in haemoglobin* at day 14 using ordinary least squares. *Hb at day 14 − Hb at baseline/Hb at baseline. **CQ AUC and weigh correlation is significant at the 0.01 level (2-tailed). Weight was excluded as a covariate. *NA* non-applicable.

## Data Availability

The evaluation of pharmacokinetics’ parameters, gender and weight as predictors of failures per treatment drug (LGM, binomial logit link); evaluation of pharmacokinetics’ parameters and weight as predictors of D3 failures per treatment drug (LGM, binomial logit link); and evaluation of pharmacokinetics’ parameters, gender and weight as predictors of time to failures per treatment drug (Cox regression, binomial logit link) are provided in the additional file.

## Abbreviations

ACT: artemisinin-based combination therapy
AE: adverse events
AL: fixed dose combination of 20 + 120 mg artemether and lumefantrine with concomitant use of primaquine
ANVISA: National Regulatory Agency
AS: CYP2D6 activity score
ASMQ: fixed dose combination of 100 + 200 mg artesunate and mefloquine with concomitant use of primaquine
AUC: area-under-the-curve
CQ: chloroquine
CI: confidence interval
CQ: chloroquine with concomitant use of primaquine
G6PD: glucose-6-phosphate dehydrogenase
GEE: generalized estimation equation
GLM: generalized linear models
Hb: haemoglobin
HPLC-MS/MS: high performance liquid chromatography-tandem mass spectrometry
LMF: lumefantrine
MQ: mefloquine
OR: odds ratio
PCR: polymerase chain reaction
RR: relative risks
SD: standard deviations
SEFAR: Equivalence and Pharmacokinetics Service
t_1/2_: half-life
WHO: World Health Organization
