# Supplementary material for: Pharmacokinetics/pharmacodynamics of chloroquine and artemisinin-based combination therapy with primaquine
Source: Malar J. 2019 Sep 23;18:325. doi: 10.1186/s12936-019-2950-4 (PMC6757423; doi:10.1186/s12936-019-2950-4)
Supplement: Supplementary file 1 — Additional file 1. Genotyping of Plasmodium vivax polymorphic loci from patients during initial infection and recurrence. Size of PCR products in base pairs (bp). #Number of patient and day of sample collection: d0—diagnosis and treatment, dX—the day of recurrence. *Allele similar to initial infection present in lower intensity. NA non-amplified. [file 12936_2019_2950_MOESM1_ESM.docx]

| Table S1: Genotyping of *Plasmodium vivax* polymorphic loci from patients during initial infection and recurrence | | | | | |  |
| --- | --- | --- | --- | --- | --- | --- |
| **ID sample#** | **MS2** | **MS6** | **MS7** | **MSP1B2** | **MSP1B10** | **Classification** |
| 11 d0 | 294 bp | 233 bp | 349 bp | 410 bp | 290 bp | Related (homologous) |
| 11 d42 | 294 bp | 233 bp | 349 bp | 410 bp | 290 bp |  |
| 20 d0 | 298 bp | 209 bp | 349 bp | 426 bp | 255 bp | Related (homologous) |
| 20 d63 | 298 bp | 209 bp | 349 bp | 426 bp | 255 bp |  |
| 24 d7 | 342 bp | 199 bp | 385 bp | NA | 255 bp | Unrelated (heterologous) |
| 24 d63 | 298 bp | 199 bp | 349 bp | 410 bp | 223 bp |  |
| 33 d0 | 298 bp | 209 bp | 349 bp | 426 bp | 255 bp | Related (similar) |
| 33 d62 | 298 bp | 209 bp | 385 bp | 426 bp | 255 bp |  |
| 35 d0 | 292 bp | 209 bp | 355 bp | 426 bp | 290 bp | Unrelated (heterologous) |
| 35 d62 | 300 bp | 257 bp | 358 bp | 426 bp | 255 bp |  |
| 43 d0 | 294 bp | 233 bp | 349 bp | 410 bp | 290 bp | Related (homologous) |
| 43 d51 | 294 pb | 233 bp | 349 bp | 410 bp | 290 bp |  |
| 50 d0 | 312 bp | 209 bp | 361 bp | 397 bp | 242 bp | Unrelated (heterologous) |
| 50 d41 | 302 bp | 209 bp | 355 bp | 410 bp | 237 bp |  |
| 53 d0 | 298 bp | 209 bp | 361 bp | 397 bp | 233 bp | Related (homologous) |
| 53 d49 | 298 bp | 209 bp | 361 bp | 397 bp | 233 bp |  |
| 60 d0 | 298 bp | 209 bp | 361 bp | 397 bp | 242bp | Unrelated (heterologous) |
| 60 d63 | 292 bp | 199 bp* | 388 bp | 397 bp | 255 bp* |  |
| 67 d0 | 294 bp | 230 bp | 349 bp | 410 bp | 290 bp | Related (homologous) |
| 67 d54 | 294 bp | 230 bp | 349 bp | 410 bp | 290 bp |  |
| 69 d0 | 298 bp | 209 bp | 349 bp | 414 bp | 223 bp | Related (homologous) |
| 69 d28 | 298 bp | 209 bp | 349 bp | 414 bp | 223 bp |  |
| 73 d0 | 292 bp | 209 bp | 358 bp | 397 bp | 255 bp | Related (homologous) |
| 73 d37 | 292 bp | 209 bp | 358 bp | 397 bp | 255 bp |  |
| 102 d0 | 298 bp | 209 bp | 349 bp | 410 bp | 223 bp | Related (homologous) |
| 102 d63 | 298 bp | 209 bp | 349 bp | 410 bp | 223 bp |  |
| 103 d0 | 294 bp | 230 bp | 349 bp | 410 bp | 290 bp | Related (homologous) |
| 103 d40 | 294 bp | 230 bp | 349 bp | 410 bp | 290 bp |  |
| 111 d0 | 300 bp | 209 bp | 355 bp | 426 bp | 237 bp | Unrelated (heterologous) |
| 111 d50 | 292 bp* | 209 bp | 349 bp | 409 bp* | 237 bp |  |
| 119 d0 | 300 bp | 209 bp | 349 bp | 408 bp | 290 bp | Related (homologous) |
| 119 d27 | 300 bp | 209 bp | 349 bp | 408 bp | 290 bp |  |
| 126 d0 | 298 bp | 209 bp | 355 bp | 410 bp | 237 bp | Related (similar) |
| 126 d47 | 298 bp | 209 bp | 355 bp | 426 bp* | 237 bp |  |
| 133 d0 | 298 bp | 209 bp | 355 bp | 410 bp | 237 bp | Related (homologous) |
| 133 d62 | 298 bp | 209 bp | 355 bp | 410 bp | 237 bp |  |
| 136 d0 | 298 bp | 209 bp | 355 bp | 426 bp | 237 bp | Related (homologous) |
| 136 d63 | 298 bp | 209 bp | 355 bp | 426 bp | 237 bp |  |
| 153 d0 | 298 bp | 209 bp | 358 bp | 397 bp | 242 bp | Related (similar) |
| 153 d63 | 292 bp | 209 bp | 358 bp | 397 bp | 242 bp |  |
| 156 d0 | 300 bp | 209 bp | 349 bp | 397 bp | 255 bp | Related (homologous) |
| 156 d63 | 300 bp | 209 bp | 349 bp | 397 bp | 255 bp |  |
| 157 d0 | 298 bp | 209 bp | 361 bp | 381 bp | 242 bp | Unrelated (heterologous) |
| 157 d63 | 298 bp | 199 bp* | 358 bp | NA | 242 bp |  |
| 160 d0 | 298 bp | 230 bp | 349 bp | 426 bp | 255 bp | Related (homologous) |
| 160 d61 | 298 bp | 230 bp | 349 bp | 426 bp | 255 bp |  |
| 162 d0 | 294 bp | 209 bp | 349 bp | 397 bp | 242 bp | Related (similar) |
| 162 d56 | 292 bp | 209 bp | 349 bp | 397 bp | 242 bp |  |
| 163 d0 | 298 bp | 209 bp | 361 bp | 405 bp | 223 bp | Related (homologous) |
| 163 d42 | 298 bp | 209 bp | 361 bp | 405 bp | 223 bp |  |
| 166 d0 | 294 bp | 230 bp | 349 bp | 392 bp | 290 bp | Related (similar) |
| 166 d57 | 294 bp | 230 bp | 349 bp | 410 bp | 290 bp |  |
| 182 d0 | 298 bp | 224 bp | 343 bp | 426 bp | 255 bp | Related (homologous) |
| 182 d21 | 298 bp | 224 bp | 343 bp | 426 bp | 255 bp |  |
| 198 d0 | 292 bp | 209 bp | 358 bp | 397 bp | 255 bp | Related (homologous) |
| 198 d62 | 292 bp | 209 bp | 358 bp | 397 bp | 255 bp |  |
| 207 d0 | 300 bp | 209 bp | 393 bp | 410 bp | 290 bp | Related (similar) |
| 207 d56 | 300 bp | 209 bp | 390 bp | 410 bp | 290 bp |  |
| 214 d0 | 298 bp | 209 bp | 352 bp | 426 bp | 264 bp | Related (homologous) |
| 214 d42 | 298 bp | 209 bp | 352 bp | 426 bp | 264 bp |  |
| 216 d0 | 298 bp | 199 bp | 427 bp | 397 bp | 327 bp | Unrelated (heterologous) |
| 216 d55 | 292 bp | 209 bp | 430 bp | 414 bp | 249 bp |  |
| 231 d0 | 298 bp | 209 bp | 355 bp | 410 bp | 290 bp | Related (homologous) |
| 231 d63 | 298 bp | 209 bp | 355 bp | 410 bp | 290 bp |  |
| 246 d0 | 298 bp | 209 bp | 352 bp | 410 bp | 223 bp | Related (homologous) |
| 246 d39 | 298 bp | 209 bp | 352 bp | 410 bp | 223 bp |  |
| 250 d0 | 298 bp | 209 bp | 352 bp | 426 bp | 264 bp | Related (similar) |
| 250 d63 | 298 bp | 233 bp | 352 bp | 426 bp | 264 bp |  |
| 251 d0 | 294 bp | 209 bp | 349 bp | 410 bp | 290 bp | Related (homologous) |
| 251 d63 | 294 bp | 199 bp | 340 bp | 410 bp | 290 bp |  |
| Number of alleles/Total | 7 | 6 | 13 | 9 | 9 | 43 |
| H*_E_* | 0.68 | 0.51 | 0.79 | 0.76 | 0.82 | 0.71 |
| Size of PCR products in base pairs (bp). #Number of patient and day of sample collection: d0 – diagnosis and treatment, dX – the day of recurrence. *Allele similar to initial infection present in lower intensity. NA – non-amplified. H*_E_* – heterozigosity expected calculated in Arlequin software. | | | | | | |
