# Supplementary material for: Pharmacokinetics/pharmacodynamics of chloroquine and artemisinin-based combination therapy with primaquine
Source: Malar J. 2019 Sep 23;18:325. doi: 10.1186/s12936-019-2950-4 (PMC6757423; doi:10.1186/s12936-019-2950-4)
Supplement: Supplementary file 2 — Additional file 2. Genotyping of CYP2C8 gene and CYP2D6 gene and predicted phenotype of CYP2D6 activity. a CYP2D6 haplotype inferred using Phase software. b Activity score and inferred phenotype of CYP2D6: AS = 1.5 or 2 − normal metabolizer fast (gNM-F); AS ≥ 2 − ultra metabolizer (gUM) (more than 2 copies of the normal allele); AS = 1 − normal metabolizer slow (gNM-S); AS = 0.5 − intermediate metabolizer (gIM); AS = 0 − poor metabolizer (gPM), according to Gaedigk et al. [20]. c Sum of AS attributed to allele 1 and 2 of CYP2D6 gene. NP not performed. [file 12936_2019_2950_MOESM2_ESM.docx]

|  | | Table S2: Genotyping of *CYP2C8* gene and *CYP2D6* gene and predicted phenotype of CYP2D6 activity | | | | | | | | | | | | | | | | | |
| --- | --- | --- | --- | --- | --- | --- | --- | --- | --- | --- | --- | --- | --- | --- | --- | --- | --- | --- | --- |
| **ID patient** | | **CYP2C8** | |  | **CYP2D6** | | | | | | | | | | | | | | |
|  |  | **G1846A** | **G2988A** |  | **G-1584C** | **C100T** | **C1023T** | **C2850T** | **G3183A** | **G4180C** | **2615_2617 del AAG** | **Haplotype^a^** | **Allele 1** | **Allele 2** | **Copy number** | **AS^b^ Allele 1** | **AS Allele 2** | **Total activity Score^c^** | **Phenotype^b^** |
| 11 | GG | | **GA** |  | **CG** | CC | CC | **TT** | GG | **CC** | AA | PV011: (4,7) | *2/*35 | *41 | 2 | 1 | 0.5 | 1.5 | gNM-F |
| 20 | GG | | GG |  | CC | **TC** | CC | CC | GG | **GC** | AA | PV020: (2,6) | *1 | *10/*10x2 | 2 | 1 | 0.5 | 1.5 | gNM-F |
| 24 | GG | | GG |  | **CG** | CC | **CT** | **TT** | GG | **CC** | AA | PV024: (4,5) | *2/*35 | *17 | 2 | 1 | 0.5 | 1.5 | gNM-F |
| 33 | **GA** | | GG |  | CC | **TC** | **TT** | **TC** | GG | **CC** | AA | PV033: (5,10) | *17 | NP | 2 | 0.5 | NP | NP | NP |
| 35 | GG | | GG |  | CC | CC | CC | CC | GG | GG | AA | PV035: (2,2) | *1 | *1 | 2 | 1 | 1 | 2 | gNM-F |
| 43 | **GA** | | GG |  | CC | **TC** | CC | CC | GG | **GC** | AA | PV043: (2,8) | *1 | *4/*4x2 | 2 | 1 | 0 | 1 | gNM-S |
| 50 | GG | | GG |  | CC | CC | CC | CC | GG | GG | AA | PV050: (2,2) | *1 | *1 | 2 | 1 | 1 | 2 | gNM-F |
| 53 | GG | | GG |  | CC | **TC** | CC | CC | GG | **GC** | AA | PV053: (2,6) | *1 | *10 | 2 | 1 | 0.5 | 1.5 | gNM-F |
| 60 | GG | | GG |  | **GG** | CC | CC | **TT** | GG | **CC** | AA | PV060: (4,4) | *2/*35 | *35xN/*2xN | 3 | 1 | 2 | 3 | gUM |
| 67 | **AA** | | GG |  | CC | **TT** | CC | CC | GG | **CC** | AA | PV067: (8,8) | *4 | *4 | 2 | 0 | 0 | 0 | gPM |
| 69 | GG | | GG |  | CC | CC | CC | CC | GG | GG | AA | PV069: (2,2) | *1 | *1 | 2 | 1 | 1 | 2 | gNM-F |
| 73 | GG | | GG |  | CG | CC | CC | **TC** | GG | **GC** | AA | PV073: (2,4) | *1 | *2/*35 | 2 | 1 | 1 | 2 | gNM-F |
| 102 | GG | | **GA** |  | CC | CC | CC | **TC** | GG | **GC** | AA | PV102: (2,7) | *1 | *41 | 2 | 1 | 0.5 | 1.5 | gNM-F |
| 103 | GG | | GA |  | **CG** | CC | CC | **TT** | GG | **CC** | AA | PV103: (4,7) | *2/*35 | *41 | 2 | 1 | 0.5 | 1.5 | gNM-F |
| 111 | GG | | GG |  | CC | CC | CC | CC | GG | GG | AA | PV111: (2,2) | *1 | *1 | 2 | 1 | 1 | 2 | gNM-F |
| 119 | GG | | GG |  | CG | CC | **CT** | **TT** | GG | **CC** | AA | PV119: (4,5) | *2/*35 | *17 | 2 | 1 | 0.5 | 1.5 | gNM-F |
| 126 | **GA** | | GG |  | CC | **TC** | CC | CC | GG | **GC** | AA | PV126: (2,8) | *1 | *4 | 2 | 1 | 0 | 1 | gNM-S |
| 133 | GG | | GG |  | CC | CC | CC | CC | GG | GG | AA | PV133: (2,2) | *1 | *1 | 2 | 1 | 1 | 2 | gNM-F |
| 136 | **GA** | | GG |  | CC | **TC** | CC | CC | GG | **GC** | AA | PV136: (2,8) | *1 | *4 | 2 | 1 | 0 | 1 | gNM-S |
| 153 | GG | | GG |  | CC | CC | CC | CC | GG | GG | AA | PV153: (2,2) | *1 | *1 | 2 | 1 | 1 | 2 | gNM-F |
| 156 | GG | | GG |  | **GG** | CC | CC | **TT** | GG | **CC** | AA | PV156: (4,4) | *2/*35 | *2/*35 | 2 | 1 | 1 | 2 | gNM-F |
| 157 | GG | | GG |  | **GG** | CC | CC | **TT** | GG | **CC** | AA | PV157: (4,4) | *2/*35 | *5 | 1 | 1 | 1 | 2 | gNM-F |
| 160 | **GA** | | GG |  | CC | **TC** | CC | CC | GG | **GC** | AA | PV160: (2,8) | *1/*1xN | *4/*4x2 | NP | 1 | 0 | 1 | gNM-S |
| 162 | GG | | GG |  | **CG** | CC | CC | **TC** | GG | **GC** | AA | PV162: (2,4) | *1 | *2/*35 | 2 | 1 | 1 | 2 | gNM-F |
| 163 | **GA** | | **GA** |  | CC | **TC** | CC | **TC** | GG | **CC** | AA | PV163: (7,8) | *41 | *4/*4x2 | NP | 0.5 | 0 | 0.5 | gIM |
| 166 | GG | | GG |  | **CG** | CC | CC | **TT** | GG | **CC** | AA | PV166: (1,4) | *2D | *2/*35 | 2 | 1 | 1 | 2 | gNM-F |
| 182 | GG | | **GA** |  | CC | CC | CC | **TC** | GG | **GC** | AA | PV182: (2,7) | *1 | *41 | 2 | 1 | 0.5 | 1.5 | gNM-F |
| 198 | GG | | GG |  | CC | **TC** | CC | CC | GG | **GC** | **CA** | PV198: (3,6) | *9 | *10 | 2 | 0.5 | 0.5 | 1 | gNM-S |
| 207 | GG | | GG |  | **CG** | CC | CC | **TC** | GG | **GC** | **CA** | PV207: (3,4) | *9 | *2/*35 | 2 | 0.5 | 1 | 1.5 | gNM-F |
| 214 | GG | | GG |  | **GG** | CC | CC | **TT** | GG | **CC** | AA | PV214: (4,4) | *2/*35 | *2/*35 | 2 | 1 | 1 | 2 | gNM-F |
| 216 | **GA** | | GG |  | CC | **TC** | CC | CC | GG | **GC** | AA | PV216: (2,8) | *1/*1xN | *4/*4x2 | 3 | 2 | 0 | 2 | gNM-S |
| 231 | **GA** | | GG |  | **GG** | **TC** | CC | **TC** | GG | **CC** | AA | PV231: (4,9) | *2/*35/*2xN | NP | 3 | 1 | NP | NP | NP |
| 246 | **GA** | | GG |  | CC | **TC** | CC | CC | GG | **GC** | AA | PV246: (2,8) | *1 | *4 | 2 | 1 | 0 | 1 | gNM-S |
| 250 | GG | | GG |  | CC | CC | CC | CC | GG | GG | AA | PV250: (2,2) | *1 | *1 | 2 | 1 | 1 | 2 | gNM-F |
| 251 | GG | | **GA** |  | **CG** | CC | CC | **TT** | GG | **CC** | AA | PV251: (4,7) | *2/*35 | *41 | 2 | 1 | 0.5 | 1.5 | gNM-F |
| a *CYP2D6* haplotype inferred using Phase software. b Activity score and inferred phenotype of CYP2D6: AS = 1.5 or 2 – normal metabolizer fast (gNM-F); AS ≥ 2 - ultra metabolizer (gUM) (more than 2 copies of the normal allele); AS = 1 – normal metabolizer slow (gNM-S); AS = 0.5 – intermediate metabolizer (gIM); AS = 0 – poor metabolizer (gPM), according to Gaedigk et al. 2008 [20]. c Sum of AS attributed to allele 1 and 2 of *CYP2D6* gene. NP - not performed. | | | | | | | | | | | | | | | | | | | |
