# Supplementary material for: Pharmacokinetics/pharmacodynamics of chloroquine and artemisinin-based combination therapy with primaquine
Source: Malar J. 2019 Sep 23;18:325. doi: 10.1186/s12936-019-2950-4 (PMC6757423; doi:10.1186/s12936-019-2950-4)
Supplement: Supplementary file 3 — Additional file 3. Evaluation of pharmacokinetics’ parameters, gender and weight as predictors of failures per treatment drug (Generalized Linear Model, binomial logit link). [file 12936_2019_2950_MOESM3_ESM.docx]

| Table S3: Evaluation of pharmacokinetics’ parameters, gender and weight as predictors of failures per treatment drug (Generalized Linear Model, binomial logit link) | | | |
| --- | --- | --- | --- |
|  | **MQ** | **CQ** | **LMF** |
| **n failures (%)** | 7 (8) | 7 (13) | 8 (14) |
|  | **OR (95% CI) p-value** | | |
| **Female** | 0.73 (0.15-3.53), p=0.7 | 2.13 (0.09-50.66), p=0.64 | 0.35 (0.06-1.94), p=0.23 |
| **Half-life (days)** | - | 1.04 (0.87-1.23), p=0.69 | - |
| **Weight** | 0.94 (0.88-1.02), p=0.13 | 0.92 (0.84-1.01), p=0.09 | 0.93 (0.83-1.03), p=0.16 |
| **AUC (mcg)** | 1 (0.99-1.01), p=0.71 | 0.99 (0.96-1.02), p=0.63 | 0.97 (0.87-1.07), p=0.52 |
