# Supplementary material for: Pharmacokinetics/pharmacodynamics of chloroquine and artemisinin-based combination therapy with primaquine
Source: Malar J. 2019 Sep 23;18:325. doi: 10.1186/s12936-019-2950-4 (PMC6757423; doi:10.1186/s12936-019-2950-4)
Supplement: Supplementary file 4 — Additional file 4. Evaluation of pharmacokinetics’ parameters and weight as predictors of D3 failures per treatment drug (Generalized Linear Model, binomial logit link). Only available to chloroquine, all males. ASMQ and AL 100% presented clearance at D3. [file 12936_2019_2950_MOESM4_ESM.docx]

| Table S4: Evaluation of pharmacokinetics’ parameters and weight as predictors of D3 failures per treatment drug (Generalized Linear Model, binomial logit link) | | | |
| --- | --- | --- | --- |
|  | **MQ** | **CQ** |  |
| **n failures (%)** | - | 3 (6) |  |
|  | **OR (95% CI), p-value** | | |
| **Weight** | - | 0.93 (0.84-1.03), p=0.18 |  |
| **AUC (mcg)** | - | 1.00 (0.99-1.02), p=0.70 |  |
| **Half-life (days)** | - | 1.05 (0.9-1.23), p=0.52 |  |
| Only available to chloroquine, all males. ASMQ and AL 100% presented clearance at D3. | | | |
