# Supplementary material for: Pharmacokinetics/pharmacodynamics of chloroquine and artemisinin-based combination therapy with primaquine
Source: Malar J. 2019 Sep 23;18:325. doi: 10.1186/s12936-019-2950-4 (PMC6757423; doi:10.1186/s12936-019-2950-4)
Supplement: Supplementary file 5 — Additional file 5. Evaluation of pharmacokinetics’ parameters, gender and weight as predictors of time to failures per treatment drug (Cox regression). [file 12936_2019_2950_MOESM5_ESM.docx]

| Table S5: Evaluation of pharmacokinetics’ parameters, gender and weight as predictors of time to failures per treatment drug (Cox regression) | | | |
| --- | --- | --- | --- |
|  | **MQ** | **CQ** | **LMF** |
| **n events (%)** | 7 (8) | 7 (8) | 8 (9) |
|  | **HR (95% CI), p-value** | | |
| **Weight** | 0.95 (0.89-1.02), p=0.18 | 0.93 (0.85-1.03), p=0.15 | 0.93 (0.86-1.02), p=0.11 |
| **AUC (mcg)** | 1 (0.99-1.01), p=0.95 | 0.99 (0.96-1.02), p=0.65 | 0.96 (0.87-1.07), p=0.46 |
| **Half-life (days)** | - | 1.02 (0.89-1.17), p=0.8 | - |
| **Gender** | 0.74 (0.14-3.83), p=0.72 | 1.87 (0.2-17.84), p=0.59 | 0.38 (0.06-2.42), p=0.31 |
