# Supplementary material for: Pharmacokinetics/pharmacodynamics of chloroquine and artemisinin-based combination therapy with primaquine
Source: Malar J. 2019 Sep 23;18:325. doi: 10.1186/s12936-019-2950-4 (PMC6757423; doi:10.1186/s12936-019-2950-4)
Supplement: Supplementary file 6 — Additional file 6. Evaluation of pharmacokinetics’ parameters as predictors of frequent (n ≥ 30) adverse event (possible and likely related to treatment) per system and drug using Generalized Estimation Equation log-binomial regression. *CQ AUC and weigh correlation is significant at the 0.01 level (2-tailed). Weight was excluded as a covariate. [file 12936_2019_2950_MOESM6_ESM.docx]

| Table S6: Evaluation of pharmacokinetics’ parameters and weight as predictors of the drop in haemoglobin* at day 14 using ordinary least squares. | | | |
| --- | --- | --- | --- |
|  | **Mefloquine (N=52)** | **Chloroquine (N=52)** | **Lumefantrine (N=53)** |
|  | **Coefficient (95%CI), p-value** | | |
| **AUC (0d-63d) (µg/ml.h)** | 0.00 (-0.24-0.33), p=0.17 | -0.02 (-0.03;-0.005), p=0.01 | 0.00 (-0.16-0.33), p=0.42 |
| **Half-life (days)** | 0.00 (0-0), p=0.87 | -0.03 (-0.1-0.04), p=0.42 | NA |
| **Weight** | 0.00 (0-0), p=0.77 | ** | 0.00 (0-0), p=0.37 |
| *Hb at day 14 − Hb at baseline/Hb at baseline  **CQ AUC and weigh correlation is significant at the 0.01 level (2-tailed). Weight was excluded as a covariate. NA= Non-Applicable | | | |
