# Supplementary material for: Pharmacokinetics/pharmacodynamics of chloroquine and artemisinin-based combination therapy with primaquine
Source: Malar J. 2019 Sep 23;18:325. doi: 10.1186/s12936-019-2950-4 (PMC6757423; doi:10.1186/s12936-019-2950-4)
Supplement: Supplementary file 7 — Additional file 7. Evaluation of pharmacokinetics’ parameters and weight as predictors of the drop in haemoglobin* at day 14 using ordinary least squares. *Hb at day 14 − Hb at baseline/Hb at baseline. **CQ AUC and weigh correlation is significant at the 0.01 level (2-tailed). Weight was excluded as a covariate. NA non-applicable. [file 12936_2019_2950_MOESM7_ESM.docx]

| Table S7: Evaluation of pharmacokinetics’ parameters as predictors of frequent (**n≥30)** adverse event (possible and likely related to treatment) per system and drug using Generalized Estimation Equation log-binomial regression. | | | | | |
| --- | --- | --- | --- | --- | --- |
| **Treatment group** |  | **Adverse event (System)**  **RR (95% CI), p-value** | | | |
|  |  | **Dermatological (pruritus)** | **Overall state** | **CNS** | **Digestive** |
| **MQ** | **AUC** | - | - | 1 (1-1), p=0.49 | 1 (1-1), p=0.61 |
|  | **Half-life** | - | - | 0.99 (0.95-1.04), p=0.71 | 1 (0.96-1.03), p=0.81 |
|  | **Weigh** | - | - | 1.00 (0.97-1.03), p=0.93 | 1 (0.97-1.03), p=0.93 |
| **CQ*** | **AUC** | 1 (0.99-1.01), p=0.73 | - | 1.01 (1-1.02), p=0.07 | 0.99 (0.98-1), p=0.06 |
|  | **Half-life** | 1.09 (1.03-1.14), p<0.01 | - | 1.02 (0.97-1.08), p=0.43 | 0.99 (0.95-1.04), p=0.73 |
| **LMF** | **AUC** | - | 0.96 (0.91-1.01), p=0.15 | - | 0.98 (0.95-1.01), p=0.24 |
|  | **Weigh** | - | 1.01 (0.98-1.04), p=0.57 | - | 0.97 (0.94-1.01), p=0.11 |
| *CQ AUC and weigh correlation is significant at the 0.01 level (2-tailed). Weight was excluded as a covariate | | | | | |
